# p16^INK4a^ Prevents Centrosome Dysfunction and Genomic Instability in Primary Cells

**DOI:** 10.1371/journal.pbio.0040051

**Published:** 2006-02-14

**Authors:** Kimberly M McDermott, Jianmin Zhang, Charles R Holst, B. Krystyna Kozakiewicz, Veena Singla, Thea D Tlsty

**Affiliations:** **1**Department of Pathology and UCSF Comprehensive Cancer Center, University of California San Francisco, San Francisco, California, United States of America; National Cancer InstituteUnited States of America

## Abstract

Aneuploidy, frequently observed in premalignant lesions, disrupts gene dosage and contributes to neoplastic progression. Theodor Boveri hypothesized nearly 100 years ago that aneuploidy was due to an increase in centrosome number (multipolar mitoses) and the resultant abnormal segregation of chromosomes. We performed immunocytochemistry, quantitative immunofluorescence, karyotypic analysis, and time-lapse microscopy on primary human diploid epithelial cells and fibroblasts to better understand the mechanism involved in the production of supernumerary centrosomes (more than two microtubule nucleating bodies) to directly demonstrate that the presence of supernumerary centrosomes in genomically intact cells generates aneuploid daughter cells. We show that loss of p16^INK4a^ generates supernumerary centrosomes through centriole pair splitting. Generation of supernumerary centrosomes in human diploid epithelial cells was shown to nucleate multipolar spindles and directly drive production of aneuploid daughter cells as a result of unequal segregation of the genomic material during mitosis. Finally, we demonstrate that p16^INK4a^ cooperates with p21 through regulation of cyclin-dependent kinase activity to prevent centriole pair splitting. Cells with loss of p16^INK4a^ activity have been found in vivo in histologically normal mammary tissue from a substantial fraction of healthy, disease-free women. Demonstration of centrosome dysfunction in cells due to loss of p16^INK4a^ suggests that, under the appropriate conditions, these cells can become aneuploid. Gain or loss of genomic material (aneuploidy) may provide the necessary proproliferation and antiapoptotic mechanisms needed for the earliest stages of tumorigenesis.

## Abstract 2

Here the authors show that aneuploidy (where a cell has an abnormal number of chromosomes) can be caused by cells having an abnormal number of centrosomes, which leads to asymmetric cell division.

## Introduction

One of the hallmarks of cancer is the accumulation of genomic abnormalities. Aneuploidy is the most frequently identified genomic abnormality in cancer and has been shown to occur early in progression, often observed in premalignant lesions. Aneuploidy is also seen in histologically normal tissue adjacent to cancer, strongly suggesting that increased dosage of oncogenic genes and decreased dosage of tumor suppressor genes may be involved in the earliest stages of tumorigenesis [1–5]. The mechanism by which aneuploidy is generated in the earliest stages of tumorigenesis is poorly understood. In his famous *Zur Frage der Entstehung maligner Tumoren* (The Origin of Malignant Tumors), published in 1914, Theodor Boveri hypothesized that multipolar mitoses cause aneuploidy [6,7]. Events resulting in supernumerary centrosomes (more than two functional microtubule nucleating bodies) can lead to multipolar spindles and, as proposed by Boveri, lead to improper segregation of the sister chromatids and generate aneuploid daughter cells. The recent discovery of supernumerary centrosomes in premalignant and malignant lesions of the breast and their correlation with aneuploidy has provided support for Boveri's hypothesis [8–11]. However, it is still unclear whether supernumerary centrosomes drive aneuploidy or simply reflect preceding consequences of aneuploidy [12].

During the normal cell cycle of mammalian cells, the centrosome is duplicated once and only once to ensure that during mitosis each daughter cell inherits one mature centrosome containing two centrioles [13]. The centrosome duplication cycle is initiated late in the G1 phase of the cell cycle. During S phase, the first physical manifestation of centrosome duplication is splitting of one centrosome into two immature centrosomes, each containing one centriole. Synthesis of the new daughter centrioles continues through S phase, using the mother centriole as a template. Beginning in early mitosis, the centrosomes migrate to opposite poles of the cell to establish the bipolar spindle, which is critical for segregation of the duplicated DNA (sister chromatids). While cyclin-dependent kinase 2 (Cdk2) activity has been shown to be required for initiation of centrosome duplication [14–17], the molecular pathways that ensure that centrosomes are duplicated once and only once within the normal cell cycle are not well understood. Coupling of the centrosome duplication and DNA replication cycles is crucial for preventing multiple rounds of centrosome duplication within a single DNA replication cycle. Uncoupling of these two cycles is one mechanism by which generation of supernumerary centrosomes may arise [18–20]. For example, in the event that the DNA replication cycle was stalled (e.g., for DNA repair), lack of inhibition of the centrosome duplication cycle may allow for the accumulation of more than two centrosomes.

In this report, we use primary cultures of human mammary epithelial cells (HMECs) to investigate the mechanism by which cells acquire supernumerary centrosomes. Further, we determine that the acquisition of supernumerary centrosomes in genomically intact cells results in aneuploid daughter cells.

## Results

### Variant HMECs Accumulate Abnormal Centrosomes with Continued Population Doublings

**Figure 1:**
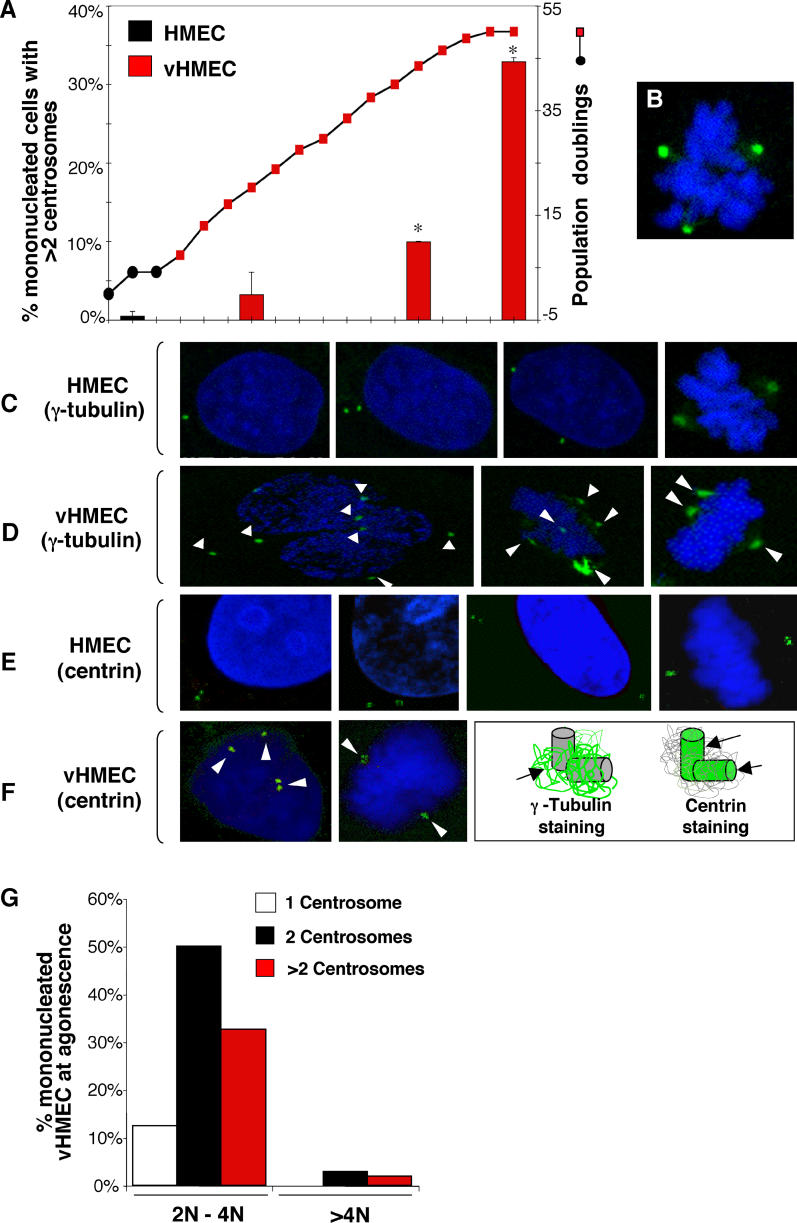
vHMECs Accumulate Mitotic and Centrosome Abnormalities (A) The solid line graph represents the in vitro growth curves of both HMECs (black circles) and vHMECs (red squares) isolated from RM16. The bar graph represents analysis of mononucleated cells containing more than two centrosomes. Centrosome number was determined by immunocytochemistry with an antibody recognizing the centrosome-associated γ-tubulin protein (excluding multinucleated cells). Cells were analyzed at multiple points along the growth curves of two different individuals (RM9 and RM16). HMEC (black) (RM9 and RM16 [less than five PD]) and vHMECs (red) analyzed at early-passage (RM9 [14 PD] and RM16 [20 PD]), late-passage (RM9 [43 PD], RM9 [65 PD]), and the agonescence (RM9 [70 PD] and RM16 [50 PD]). *Statistical significance (*p* < 0.005) based on comparison of HMECs to vHMECs. (B) Example of a late-passage vHMEC with a tripolar mitotic metaphase. Examples of centrosomes of HMECs (C and E) and vHMECs (D and F) detected by immunocytochemistry with antibodies recognizing the centrosome-associated γ-tubulin (C and D) and centrin (E and F) proteins. Examples of HMECs representing normal centrosomes during the G1 phase of the cell cycle (C and E, first panel), during the S and G2 phases of the cell cycle (C and E, second panel), during the M phase with centrosomes migrating to opposite poles of the cell (C and E, third and fourth panels), and vHMECs containing cells with more than two centrosomes (D and F, arrowhead). (G) Agonescent vHMECs (RM16 [47 and 50 PD]) were stained with an antibody recognizing γ-tubulin and with PI (DNA counterstain), and the DNA content of each nucleus was measured by quantitative immunofluorescence microscopy. Cells were classified as having 2N to 4N (diploid) or more than 4N (polyploidy) DNA content. The centrosome number (γ-tubulin signal) of each cell was linked to that individual nucleus. Analysis included 150 to 250 cells.

HMECs comprise the majority of cells that expand in culture from mammary tissues obtained from healthy women [21]. When grown in vitro under standard conditions, these cells proliferate for a limited time (five to 20 population doublings [PD]) before reaching a proliferation barrier termed *selection* (Figure 1). A small subpopulation of variant cells (vHMECs) has the ability to proliferate an additional 20 to 70 PDs beyond selection (Figure 1A), upon which they enter a phase termed *agonesence,* where they accumulate genomic abnormalities, including aneuploidy, polyploidy, structural changes (deletions/translocations) and telomeric associations [22]. These genomically unstable vHMECs also contain abnormal mitotic metaphases, such as tripolar mitoses (Figure 1B), suggesting that the genomic instability seen in vHMECs is, in part, due to centrosome abnormalities.

To determine if centrosome abnormalities are present in vHMECs, we examined the centrosomes of these cells using an antibody that recognized γ-tubulin, a component of pericentriolar material, and an antibody that recognized centrin, a component of centrioles (Figure 1F, inset) [23]. We focused on the study of mononucleated cells with more than two centrosomes to identify events that generate more than two centrosomes in genomically intact diploid cells. Mononucleated cells from two different reduction mammoplasties (RM9 and RM16) were analyzed to control for interindividual variations. HMECs were analyzed prior to the proliferation barrier, and vHMECs were analyzed early and late along their respective growth curves as indicated by the line graph (Figure 1A), which represents the growth curve of cells isolated from a single reduction mammoplasty (RM16). The incidence of HMECs with more than two centrosomes is negligible (average of 1%) (Figure 1A, 1C, and 1E). Similarly, the average fraction of early-passage vHMECs with more than two centrosomes was not statistically different (*p* = 0.07) than that seen in HMECs (Figure 1A). As illustrated by the standard deviation seen in early-passage vHMECs (Figure 1A), there are minor differences between individuals in the fraction of mononucleated cells with more than two centrosomes (ranging from 1% to 5%). In contrast, mononucleated vHMECs analyzed at late passage and agonescence contained an average of 10% and 32% of cells with more than two centrosomes, respectively (Figure 1A, 1D, and 1F), which correlated with the increase in genomic instability seen in late-passage and agonescent vHMECs [22].

To validate that the mononucleated vHMECs with more than two centrosomes at agonescence are not of polyploid DNA content, we directly determined the DNA content of each cell containing more than two centrosomes. We performed quantitative fluorescence on cells stained with propidium iodide (PI) (DNA content) and an antibody that recognized γ-tubulin (centrosome number). As shown in Figure 1G, the majority (94%) of the mononucleated vHMECs with more than two centrosomes had a diploid DNA content, demonstrating that mononucleated vHMECs with more than two centrosomes are not generated as a result of polyploidization.

### vHMECs Acquire Supernumerary Centrosomes following Inhibition of DNA Synthesis

**Figure 2:**
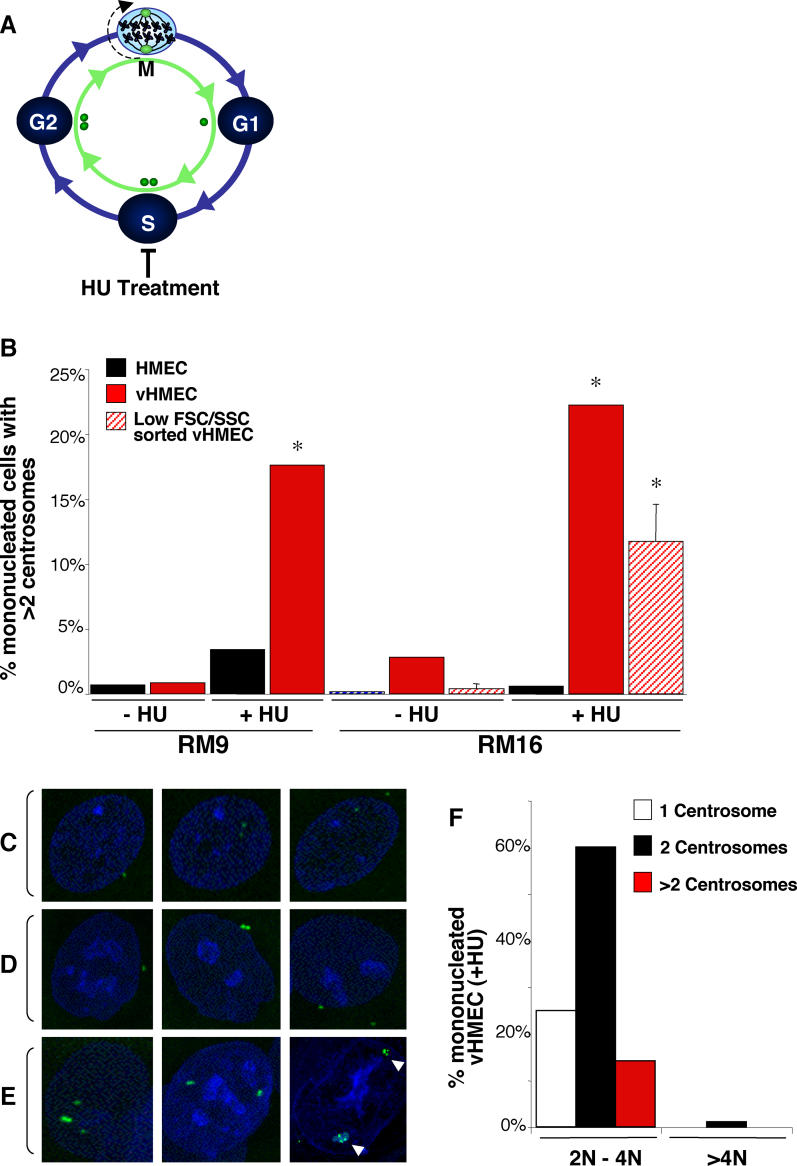
Centrosome Duplication Is Uncoupled from DNA Replication in vHMECs (A) Schematic of the centrosome duplication cycle (interior, green) in relation to the DNA replication cycle (exterior, blue). Each daughter cell inherits one copy of the DNA and one centrosome (G1 phase of the cell cycle). Centrosomes (small, green circles) begin duplication at the same point as when DNA replication is initiated (S phase of the cell cycle). Following duplication and maturation of the centrosome, the two centrosomes separate and migrate to opposite poles during early M (mitosis). (B) Analysis of mononucleated cells with more than two centrosomes in HMECs (black: RM9 [5 PD], RM16 [less than 4 PD]), early-passage vHMECs (red: RM9 [21 PD], RM16 [7 PD]), and low FSC/SSC sorted vHMECs (blue: RM16 [17 and 30 PD]) untreated (−HU) or exposed to HU (+HU). (C–E) Examples of normal centrosome numbers in HMECs (C) and vHMECs (D) and more than two centrosomes in vHMECs (E). (F) Early-passage vHMECs (RM9 [14 PD] and RM16 [17 PD]) that were exposed to HU were stained with an antibody recognizing γ-tubulin and with PI (DNA counterstain), and the DNA content of each nucleus was measured by quantitative immunofluorescence microscopy. Cells were classified as having 2N to 4N (diploid) or more than 4N (polyploidy) DNA content. The centrosome number of each cell was correlated to the DNA content of that cell. Analysis included 100 to 200 cells (excluding binucleated cells). *Statistical significance (*p* < 0.005) based on comparison of −HU and +HU experiments.

One mechanism by which excessive duplication of centrosomes can arise is due to uncoupling of the centrosome duplication and DNA replication cycles. To investigate if these two cycles are coupled in HMECs and vHMECs, we exposed the cells to a transient inhibition of DNA synthesis as previously described [17,24,25]. Exposure to hydroxyurea (HU), a reversible inhibitor of DNA synthesis, allows the cells to transition into S phase of the cell cycle but prevents them from progressing through S phase (Figure 2). In cells with coupled centrosome duplication and DNA replication cycles, centrosome duplication (generating two centrosomes) occurs; however, centrosome reduplication or generation of supernumerary centrosomes (generating more than two centrosomes) is prevented.

HMECs and vHMECs from RM9 and RM16 were exposed to 4 mM HU for 48 h and were subsequently analyzed by immunocytochemistry with anti–γ-tubulin antibody for centrosome number. Exposure of HMECs to HU arrested DNA synthesis but resulted in no statistically significant (*p* = 0.60) increase in the fraction of mononucleated cells with more than two centrosomes compared to the untreated HMEC population (Figure 2B, black bars, and 2C). In contrast, early-passage vHMECs exposed to HU had a dramatic increase in cells with more than two centrosomes. Untreated early-passage vHMECs contained a negligible background of cells with more than two centrosomes (−HU: average of 2%), whereas these same cells exposed to HU generated a striking increase in mononucleated cells with more than two centrosomes (+HU: RM9 and RM16, 18% and 22%, respectively; Figure 2B, red bars, and 2E). While the majority of the HU-exposed vHMECs with more than two centrosomes contained three or four centrosomes, a small fraction (2%) containing more than four centrosomes were also observed (Figure 2E, closed arrowhead). Analysis of the number of centrosomes following exposure to HU for longer periods of time (72, 96, and 144 h) demonstrated no statistically significant increase (*p* = 0.62, *p* = 0.729, and *p* = 1.00, respectively) in the fraction of vHMECs with more than two centrosomes as compared to vHMECs exposed to HU for 48 h. Longer exposure to HU also did not result in an increase in the fraction of cells (average of 2%) containing more than four centrosomes.

Two additional experiments assured us that the mononucleated cells with more than two centrosomes did not represent a polyploid population. vHMECs were sorted by flow cytometry for low forward scatter and side scatter (low FSC/SSC) and were analyzed for DNA content by staining with PI. PI staining demonstrated that the low FSC/SSC sorted population is enriched for cells containing a 2N DNA content and contains a reduced polyploid population, ranging from 0.6% to 1.5%. Treatment of low FSC/SSC vHMEC population (RM16) for 48 h with HU resulted in an average of 12% of the mononucleated cells with more than two centrosomes (Figure 2B, blue bars). This represented a statistically significant (*p* < 0.005) increase over the average 0.4% observed in the untreated, low FSC/SSC sorted population.

In the second experiment, we performed quantitative fluorescence analysis to directly determine the DNA content of each individual HU-exposed, early-passage vHMEC as described above. As shown in Figure 2F, 100% of the mononucleated, HU-exposed early-passage vHMECs with more than two centrosomes had a diploid DNA content. Taken together, these results demonstrate that the generation of more than two centrosomes following inhibition of DNA synthesis is not a result of polyploidization.

### Loss of p16^INK4a^ Plays a Causal Role in Centrosome Dysfunction in HMECs

Little is known about the mechanisms that prevent acquisition of more than two centrosomes. A distinguishing characteristic of vHMECs is loss of p16I^NK4a^ expression due to promoter hypermethylation [26–28], which, as described here, is correlated with the ability to acquire centrosomal abnormalities in vHMEC. Furthermore, p16INK4a is known to be an upstream regulator of Cdk2 activity, which is required to initiate both centrosome duplication and DNA replication [14–17,29], making it a plausible candidate for control of centrosome regulation.

**Figure 3:**
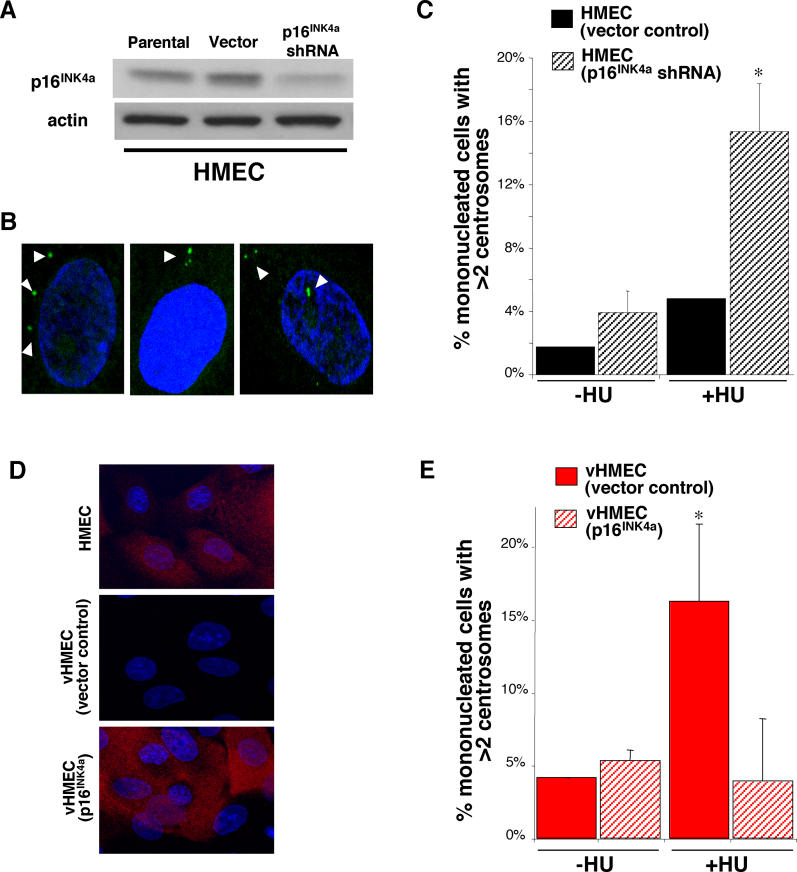
Loss of p16^INK4a^ Uncouples the Centrosome Duplication and DNA Replication Cycles in HMECs (A) Western blot analysis of p16^INK4a^ expression in HMECs parental, infected with vector-only (vector), or shRNA directed against p16^INK4a^ (p16^INK4a^ shRNA). (B, arrowhead) Examples of more than two centrosomes in HU-exposed HMECs (p16^INK4a^ shRNA). (C) HMECs infected with vector-only (black: RM9 [6 and 7 PD]) or p16^INK4a^ shRNA (gray: RM9 [7 and 8 PD]) were untreated (−HU) or exposed to HU (+HU). (D) Expression levels of p16^INK4a^ (red) in HMECs, vHMECs transfected with vector-only or p16^INK4a^. (E) vHMECs infected with vector-only (red: RM15 [33 to 37 PD]) or p16^INK4a^ (gray: RM15 [33 to 37 PD]) were untreated (−HU) or exposed to HU (+HU). Centrosome number was determined by immunocytochemistry with an antibody recognizing the centrosome-associated γ-tubulin protein. Analysis included at least 100 cells. *Statistical significance (*p* < 0.005) based on comparison of −HU and +HU experiments.

To assess the role of p16^INK4a^ in centrosome regulation, we inhibited its expression in HMECs and exposed these cells to HU as described earlier. The *p16^INK4a^* gene was inhibited through expression of a short hairpin RNA (shRNA) complementary to *p16^INK4a^* [30]. Western blot analysis and immunocytochemistry demonstrated approximately 50% suppression of p16*^INK4a^* protein expression in HMECs (Figure 3A). Following HU exposure, an average of 15% of early-passage p16^INK4a^-suppressed HMECs had more than two centrosomes (Figure 3B; Figure 3C, gray bars). This increased value was statistically significant compared to untreated cells (*p* < 0.005). Similar to parental HMECs, the vector-only and vector containing an shRNA to green fluorescent protein (GFP) control populations contained no statistically significant (*p* = 0.74 and *p* = 1.00) increase in mononucleated cells with more than two centrosomes when exposed to HU under identical conditions (Figure 3C, black bars).

Wild-type p16^INK4a^ was reexpressed in vHMECs to determine if it can rescue the described centrosome dysfunction. vHMECs were transfected with p16^INK4a^ and then exposed to HU 8 h after transfection. Expression levels of exogenous p16^INK4a^ in both the presence or absence of HU were similar to physiologic levels seen in HMECs (Figure 3D). Cell cycle analysis demonstrated that expression of p16^INK4a^ under these conditions did not alter cell cycle progression as compared to vector control (data not shown). Cells were coimmunostained with antibodies to detect p16^INK4a^ and γ-tubulin. HU exposure of p16^INK4a^-positive vHMEC arrested them in S phase but resulted in no statistically significant (p = 0.823) increase in the fraction of mononucleated cells with more than two centrosomes as compared to the untreated p16^INK4a^-positive vHMEC population (Figure 3E).

### p16^INK4a^ Prevents Centriole Pair Splitting during S Phase Arrest

**Figure 4:**
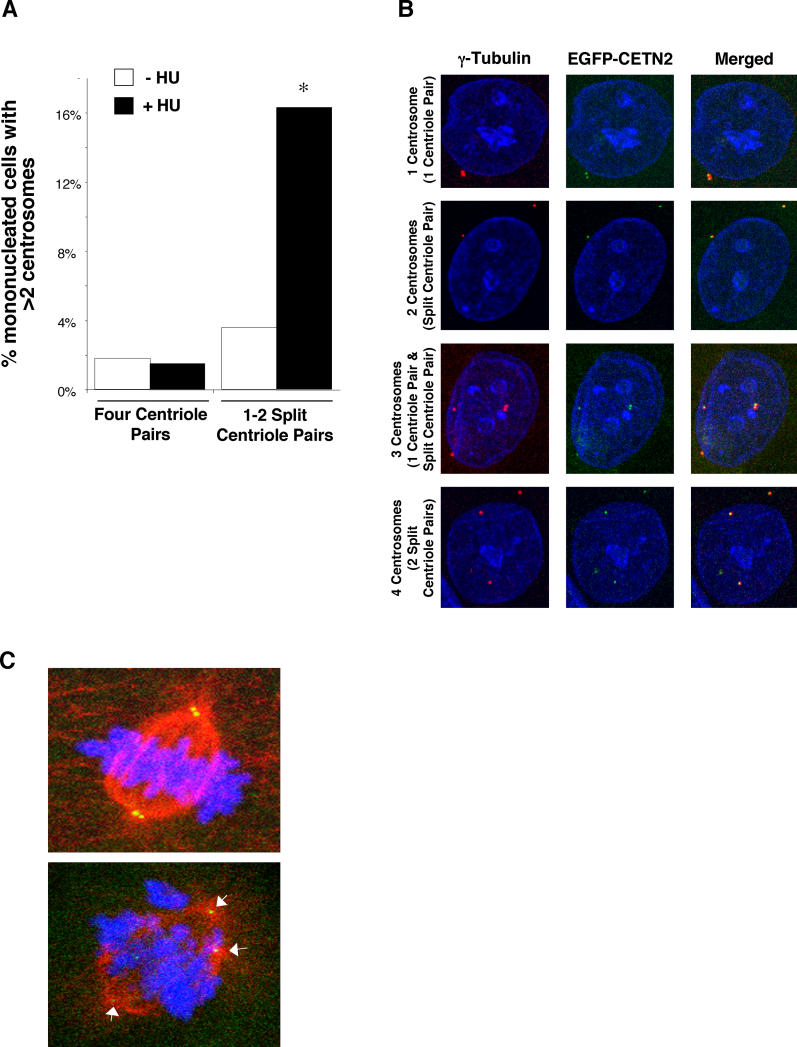
Generation of More Than Two Centrosomes in vHMECs following S Phase Arrest Is Due to Centriole Pair Splitting (A and B) Analysis of the centriole number in of the supernumerary centrosomes of early-passage vHMECs (RM15 [19 PD]) that express EGFP-CETN2 (centriole marker, green) untreated (−HU) or exposed to HU (+HU). Centrosome number was determined by immunocytochemistry with an antibody recognizing the centrosome-associated γ-tubulin protein (centrosome marker, red). Analysis included at least 100 cells (excluding binucleated cells). *Statistical significance (*p* < 0.005) based on comparison of −HU and +HU experiments. (B) Examples of HU-exposed vHMECs with one centrosome (containing a pair of centrioles), two centrosomes (each containing one centriole), three centrosomes (one of the centrosomes contains a pair of centrioles and two of the centrosomes have only one centriole), and four centrosomes (each containing one centriole). (C) Examples of HU-exposed and released vHMECs that express EGFP-CETN2 (green) and have been stained with a γ-tubulin antibody that recognizes microtubule spindles (red) that have two centrosomes, each containing two centriole (top), and supernumerary centrosomes, each containing one centriole (bottom). Supernumerary centrosomes with one centriole (arrowhead) can nucleate microtubules to form a multipolar spindle apparatus.

Loss of p16^INK4a^ activity in genomically intact diploid human cells following a transient S phase arrest generates three or four centrosomes. The rare observance of more than four centrosomes suggested that loss of p16^INK4a^ activity does not permit uncontrolled centrosome duplication under these conditions. This difference prompted us to more closely examine the supernumerary centrosomes of the vHMECs during S phase arrest. Early-passage vHMECs expressing the centriolar protein enhanced GFP (EGFP)-human centrin 2 (CETN2) were exposed to HU for 48 h and subsequently analyzed by immunocytochemistry with an antibody that recognizes the γ-tubulin protein to determine centriole number per centrosome. As described above, analysis of γ-tubulin staining demonstrated that the vHMECs (EGFP-CETN2) exposed to HU resulted in an increase in the fraction of mononucleated cells with more than two centrosomes as compared to the untreated vHMECs (EGFP-CETN2) population (Figure 4A and 4B). Analysis of the centriole number in each of the supernumerary centrosomes demonstrated that a statistically significant (*p* < 0.01) fraction of the supernumerary centrosomes contained only one centriole (Figure 4A and 4B).

Early-passage vHMECs (EGFP-CETN2) were exposed to HU for 48 h and were subsequently released from HU exposure and allowed to reenter the cell cycle for 7 to 8 d. The vHMEC (EGFP-CENT2) HU-exposed and released cells were analyzed by immunocytochemistry with an antibody that recognizes the γ-tubulin protein to determine if the pericentriolar bodies containing a single centriole could nucleate multipolar spindles. Results demonstrate that the described pericentriolar bodies containing only one centriole are able to act as functional centrosomes and nucleate microtubules during mitosis (Figure 4C). Therefore, for the purposes of this manuscript, these pericentriolar bodies that each contain only one centriole are referred to as supernumerary centrosomes.

### Acquisition of Supernumerary Centrosomes Due to Loss of p16^INK4a^ Activity Correlates with the Production of Aneuploid Daughter Cells

**Figure 5:**
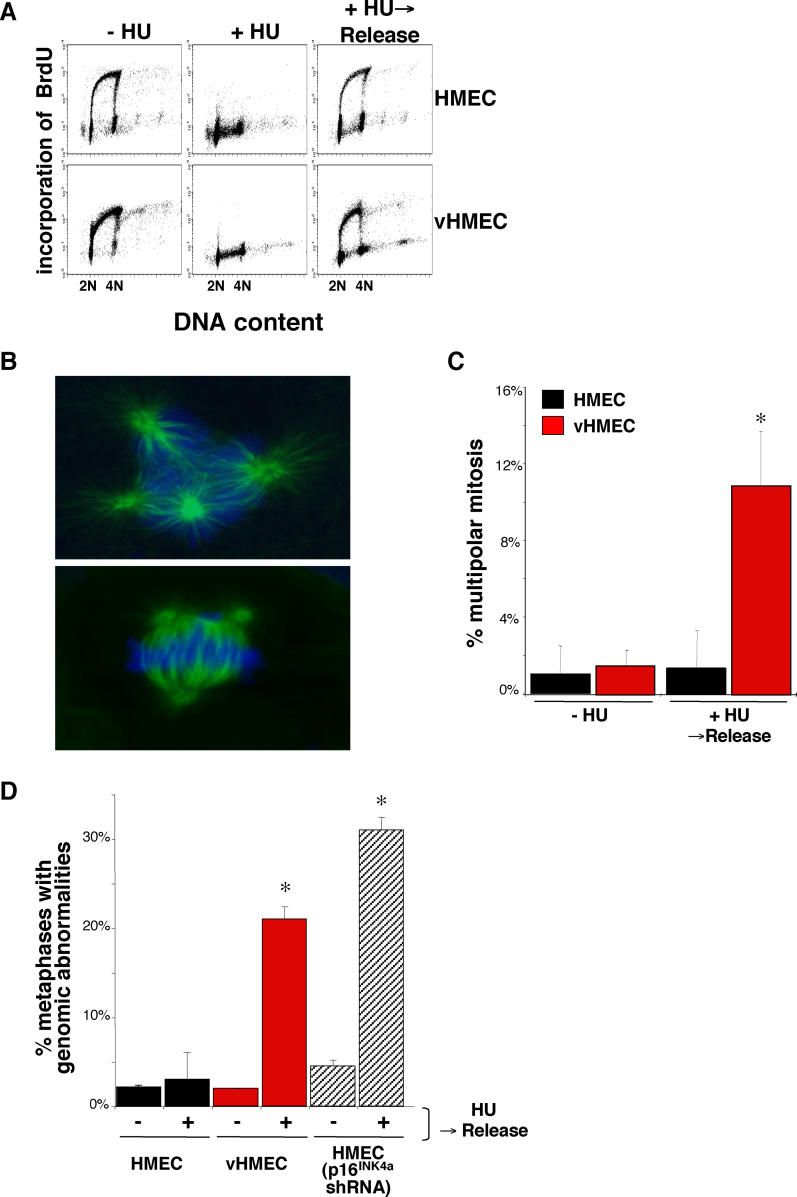
Supernumerary Centrosomes Produce Multipolar Spindles and Correlate with Aneuploidy (A) BrdU incorporation of HMECs (top) and vHMECs (bottom). Cell cycle analysis were performed using BrdU incorporation for cells that were untreated (−HU), treated for 48 h with HU (+HU), or treated for 48 h with HU, followed by release from HU treatment for 7 to 8 d (+HU → release). DNA replication resumes following release from 48 h of HU treatment. (B) Microtubule nucleation sites were determined by immunocytochemistry with an antibody recognizing γ-tubulin. (C) Analysis of HMECs (black: RM9 and RM26 [less than 4 PD]) and vHMECs (red: RM15 [25 PD], RM16 [13 PD]) for multipolar mitosis in untreated (−HU) or exposed to HU followed by release from HU treatment (+HU → release). (D) Analysis of HMECs (black: RM9 and RM15 [less than 5 PD]) and vHMECs (red: RM9 [21 PD], RM16 [17 PD]), HMECs infected with p16^INK4a^ shRNA (gray) for genomic abnormalities in untreated (−HU) or exposed to HU followed by release from HU treatment (+HU → release). Types of chromosomal abnormalities represented include aneuploidy, structural abnormalities, and telomeric associations. Analysis included at least 100 metaphases. *Statistical significance (*p* < 0.005) based on comparison of −HU and +HU → release experiments.

To test if supernumerary centrosomes drive genomic instability in vHMECs, we generated more than two centrosomes in early-passage vHMECs by transient exposure to HU. Removal of the HU released the cells from inhibition of the DNA replication cycle and allowed the cells to resume cell cycle progression (Figure 5A, bottom). HU-exposed and released HMECs and vHMECs resumed cell cycle progression similar to untreated HMECs and vHMECs (Figure 5A). Notably, exposure to HU did not result in an increased fraction of cells with polyploid DNA content. To test if HU-exposed and released early-passage vHMECs progress through mitosis with multipolar spindles, we immunostained microtubules with an antibody that recognizes γ-tubulin. Early-passage vHMECs exposed to and subsequently released from HU contained an average of 11% mitotic cells with more than two microtubule nucleation sites (Figure 5B and 5C, red bars). This was a statistically significant increase (*p* = 0.01) compared to that of untreated vHMECs. HMECs exposed to and subsequently released from HU did not have a statistically significant increase (*p* = 1.00) in the fraction of multipolar mitosis compared to untreated HMECs (Figure 5C, black bars). To determine if HU-exposed and subsequently released early-passage vHMECs have acquired genomic abnormalities, their karyotypes were analyzed and an average of 21% were found to have genomic abnormalities (Figure 5D, red bars). Specifically, we observed that 19% of these had gained or lost one or two chromosomes (aneuploidy) (19 of 100 metaphases) and the remaining 2% included a structural abnormality (one of 100 metaphases) and a telomeric association (one of 100 metaphases). In contrast, early-passage vHMEC populations not exposed to HU contained only 2% metaphases with genomic abnormalities. Thus, the fraction of HU-exposed and subsequently released vHMECs with genomic abnormalities was statistically significant (*p* < 0.005) compared to untreated vHMECs. Notably, cells observed to have genomic abnormalities contained a near diploid, but not polyploidy, DNA content (chromosome number between 44 and 48). This further supports our conclusion that, in these cell populations, more than two centrosomes are generated in a genomically intact diploid cell rather than by polyploidization.

To control for the nonspecific effects of HU on generating aneuploidy, we also performed the described HU exposure and release on HMECs. Because HMECs did not generate a significant number of cells with more than two centrosomes during HU treatment, any increase in genomic abnormalities after their release from inhibition of DNA replication would be due to nonspecific effects of HU exposure. HMECs exposed to and released from HU demonstrated a transient inhibition of DNA synthesis (Figure 5A, top) similar to that obtained in vHMECs under identical conditions (Figure 5A, bottom). However, in HU-exposed and subsequently released HMECs, less than 3% of the cells contained more than two centrosomes, and no significant increase (*p* = 1.00) in the number of metaphases with genomic abnormalities were detected (Figure 5D, black bars). These results strongly support the conclusion that the increase in aneuploidy seen in vHMECs following transient inhibition of DNA synthesis is due to supernumerary centrosomes.

We also performed the described HU exposure and release on HMECs with p16^INK4a^ shRNA. HU-exposed and subsequently released parental HMECs had no significant increase in the fraction of cells with more than two centrosomes and no significant increase in the number of genomic abnormalities (average of 3%, *p* = 1.00) as compared to untreated controls (Figure 5D, gray bars). In contrast, HMECs infected with p16^INK4a^ shRNA that were exposed and subsequently released from HU had a significant increase in both the fraction of cells with more than two centrosomes and aneuploidy (average of 31%, *p* < 0.005) following exposure to HU compared to untreated cells (Figure 5D, gray bars).

### Supernumerary Centrosomes Lead to the Production of Aneuploid Daughter Cells

**Figure 6:**
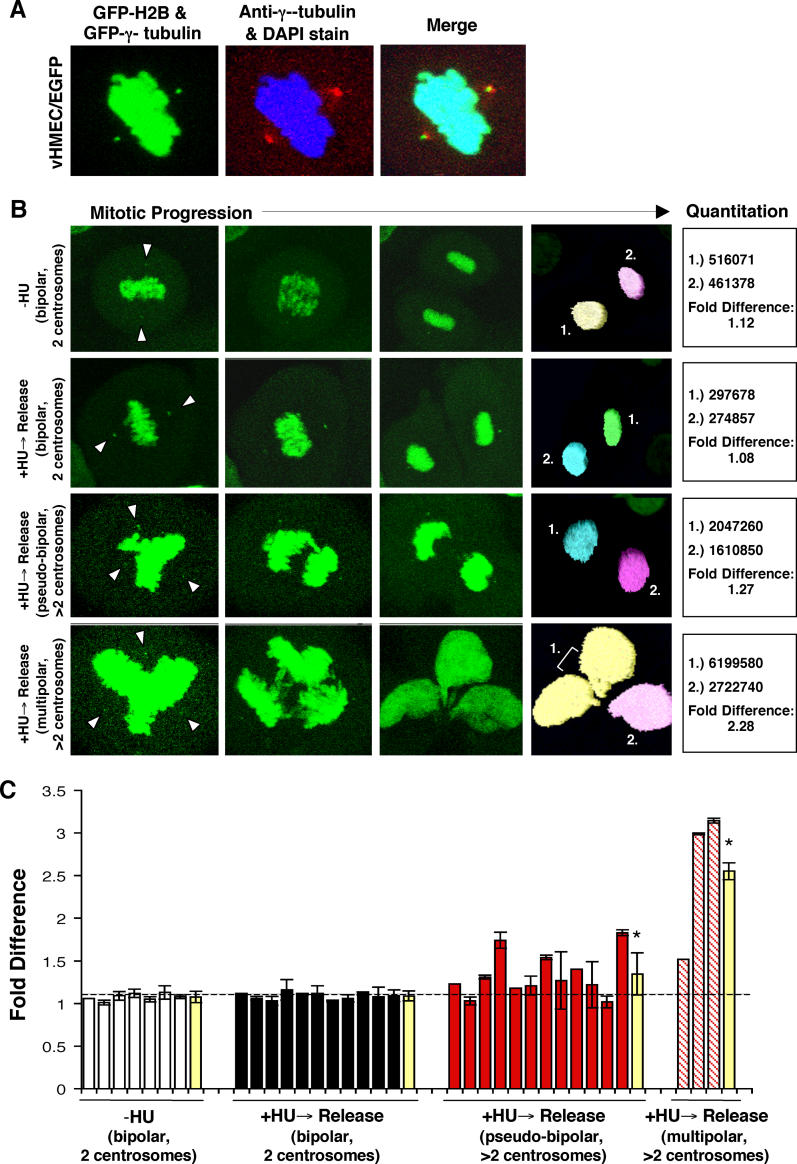
Supernumerary Centrosomes Play a Causal Role in the Production of Aneuploid Cells (A) Early-passage vHMECs (RM18, 13–33 PD) that express EGFP–γ-tubulin (green) and EGFP-H2B (green) were stained with antibody recognizing the centrosome associated γ-tubulin protein (red) and with DAPI to localize DNA (blue). The merged image demonstrates colocalization of EGFP–γ-tubulin with the centrosome and EGFP-H2B with the DNA. (B) Examples of the mitotic progression of cells that divide into two nuclei with two centrosomes (−HU and +HU → release; bipolar, two centrosomes), that divide into two nuclei with more than two centrosomes (+HU → release; bipolar, more than two centrosomes), and that divide into greater than two nuclei with more than two centrosomes (+HU → release; multipolar, with more than two centrosomes). Arrowhead points to the EGFP–γ-tubulin signal (centrosomes). The EGFP-H2B signal was selected (pastel-colored nuclei) and the total signal intensity was quantitated. Determining the fold difference (EGFP-H2B signal intensity of daughter cells 1/EGFP-H2B signal intensity of daughter cells 2) allowed us to determine whether cells had segregated their DNA equally (fold difference close to 1.00) or unequally (fold difference >1.00). (C) Bar graph of the individual (white, black, red, and red stripe) and mean (yellow) fold differences in EGFP-H2B signals intensity between daughter cells. Standard deviations represent analysis of up to ten time frames per mitotic event. The dashed line represents the average mean fold difference (1.08) of the normal mitosis (−HU and +HU → release; bipolar, two centrosomes). *Statistical significance (*p* < 0.05) based on comparison of the mean fold difference (yellow bars) of cells completing mitosis with two centrosomes (−HU and +HU → release; bipolar, two centrosomes) compared to the individual and mean fold differences of cells completing mitosis with more than two centrosomes (+HU → release; bipolar or multipolar, more than two centrosomes).

While these data described above strongly suggest that supernumerary centrosomes play a causal role in genomic instability, they still represent a correlation between supernumerary centrosomes and aneuploidy. If supernumerary centrosomes play a causal role in the generation of aneuploid daughter cells, we predict that time-lapse microscopy of cells with more than two centrosomes will lead to aneuploidy following mitosis. Early-passage vHMECs were stably infected with EGFP–γ-tubulin (centrosome marker) and EGFP-H2B (a quantifiable marker of DNA [31,32]) (vHMEC/EGFP; Figure 6A). EGFP was used as the fluorescent marker for both of these two proteins because they localize to distinct cellular organelles, and this enabled us to image the two fusion proteins with the same laser to minimize photobleaching and phototoxicity. Expression of EGFP–γ-tubulin and EGFP-H2B in the vHMECs did not alter the length of the cell cycle or the length of mitosis as compared to uninfected vHMECs or vHMECs infected with control vectors (data not shown). We generated more than two centrosomes in early-passage vHMECs expressing the EGFP fusion proteins by transient exposure to HU. The cells were released from HU, and the EGFP-H2B signal was quantified in daughter cells following mitosis.

In the control population of early-passage vHMECs expressing the EGFP fusion proteins (−HU), cells that entered mitosis with two centrosomes produced a bipolar mitotic division resulting in two daughter cells, with each having one nucleus and one centrosome (Figure 6B, first row). Analysis of the DNA content (EGFP–γ-tubulin signal intensity) of each daughter cell demonstrated a mean fold difference of 1.08. Cells exposed to and subsequently released from HU (+HU → release) that entered mitosis with two centrosomes also produced a bipolar mitotic division that resulted in two daughter cells, with each having one nucleus and one centrosome (Figure 6B, second row, and Video S1). Analysis of the DNA content of these daughter cells demonstrated a similar mean fold difference of 1.09. Cells exposed to and subsequently released from HU (+HU → release) that entered mitosis with more than two centrosomes resulted in two types of mitotic division. In the first type, the cells with more than two centrosomes produced a pseudo-bipolar mitotic division that resulted in two daughter cells, with each having one nucleus (Figure 6B, third row, and Video S2). The cells with more than two centrosomes that divided their DNA content into two daughter cells, each with one nucleus, had a mean fold difference of 1.33. In the second type, the cells with more than two centrosomes had a multipolar mitotic division that produced two or three daughter cells, each containing one, two, or, in some cases, more than two nuclei (Figure 6B, fourth row, and Video S3). Analysis of the DNA content between these resultant daughter cells demonstrated a mean fold difference of 2.55. The mean fold differences of the pseudo-bipolar and multipolar mitotic divisions with more than two centrosomes were statistically significant (*p* < 0.05) as compared to the bipolar mitotic divisions with two centrosomes (Figure 6C, yellow bars). These data strongly support the conclusion that the generation of supernumerary centrosomes in diploid cells plays a causal role in the generation of aneuploid daughter cells.

### Loss of p16^INK4a^ Plays a Causal Role in Centrosome Dysfunction and the Subsequent Generation of Aneuploid Daughter Cells in Multiple Cell Types

**Figure 7:**
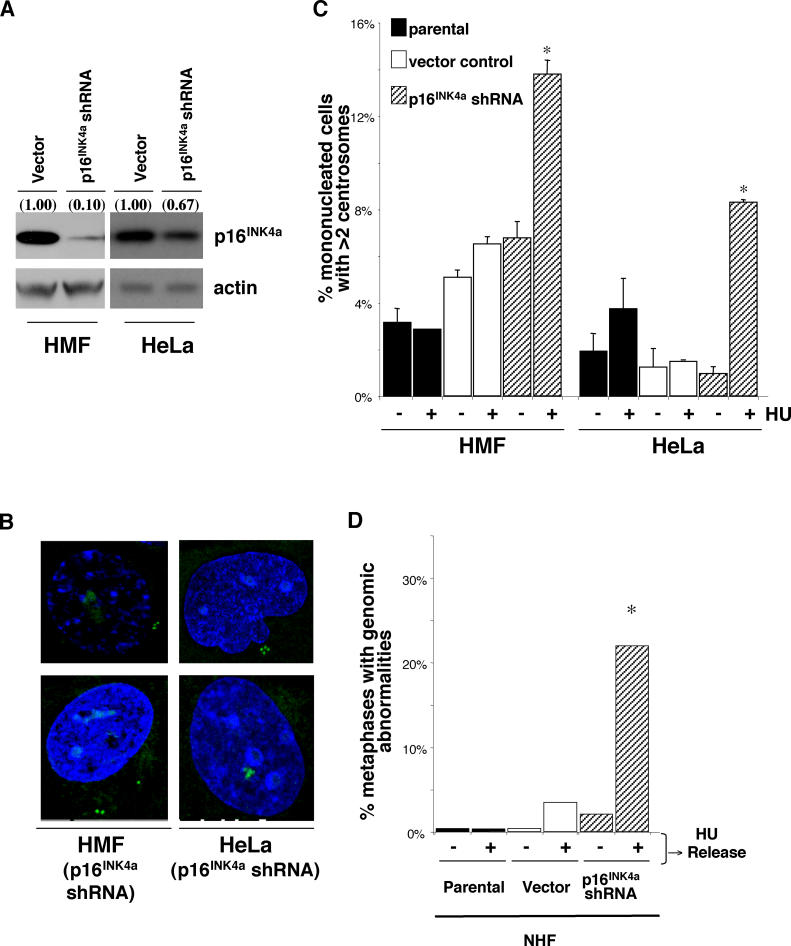
Loss of p16^INK4a^ Results in Centrosome Dysfunction and the Subsequent Generation of Aneuploid HMF and HeLa Cells (A) Western blot analysis of p16^INK4a^ expression in HMF and HeLa cells infected with vector-only (vector) or shRNA directed against p16^INK4a^ (p16^INK4a^ shRNA). (B) Examples of more than two centrosomes in HMF (p16^INK4a^ shRNA) and HeLa (p16^INK4a^ shRNA) cells. Centrosome number was determined by immunocytochemistry with an antibody recognizing the centrosome-associated γ-tubulin protein. (C) Analysis of parental HMF (RM9 [1 PD], RM21 [3 PD]) and HeLa cells (black) and HMF and HeLa cells infected with vector-only (HMF: RM9 [7 PD], RM21 [11 PD]) (white) or HMF and HeLa cells infected with p16^INK4a^ shRNA (HMF: RM9 [6 PD], RM21 [7 PD]) (gray) containing mononucleated cells with more than two centrosomes. Cells were untreated (−HU) or exposed to HU (+HU). Analysis included more than two HMF and HeLa cells. *Statistical significance (*p* < 0.005) based on comparison of −HU and +HU experiments.

To determine if the p16^INK4a^-related centrosomal dysfunction described above extends to coupled cell types other than HMECs, we suppressed expression of p16^INK4a^ in human mammary fibroblasts (HMFs), newborn dermal foreskin fibroblasts (NHFs), and the “classically coupled” HeLa cells with p16^INK4a^ shRNA. Western blot analysis and immunocytochemistry demonstrated that stable expression of p16^INK4a^ shRNA resulted in an approximately 90% suppression of p16^INK4a^ protein expression in both HMFs and NHFs and a 33% suppression in HeLa cells (Figure 7A, representative data shown for HMF and HeLa cells). HU-exposed, p16^INK4a^-suppressed HMFs, NHFs, and HeLa cells contained an average of 14%, 11%, and 8%, respectively, mononucleated cells with more than two centrosomes. This represented a statistically significant (*p* = 0.02, *p* = 0.04, *p* = 0.01, respectively) increase in cells with more than two centrosomes compared to untreated cells (Figure 7B and 7C). Following HU exposure, HMF, NHF, and HeLa parental (*p* = 1.00, *p* = 0.70, *p* = 0.44, respectively) and vector-only control (*p* = 0.60, *p* = 0.27, *p* = 1.00, respectively) populations had no statistically significant increase in cells with more than two centrosomes (Figure 7C). To determine if loss of p16^INK4a^ is causal for the supernumerary centrosome-related aneuploidy described in HMECs, we also performed the described HU exposure and release on NHFs that were infected with p16^INK4a^ shRNA. HU-exposed and subsequently released parental NHFs and NHFs infected with vector-only had no significant increase in the fraction of cells with more than two centrosomes and no significant increase in the number of genomic abnormalities as compared to untreated controls (Figure 7D). In contrast, NHFs infected with p16^INK4a^ shRNA that were exposed and subsequently released from HU had a significant increase (*p* < 0.05) in both the fraction of cells with more than two centrosomes and aneuploidy following exposure to HU compared to untreated controls (Figure 7D).

### Loss of p16^INK4a^ Results in Unregulated Kinase Activity during Inhibition of DNA Replication

**Figure 8:**
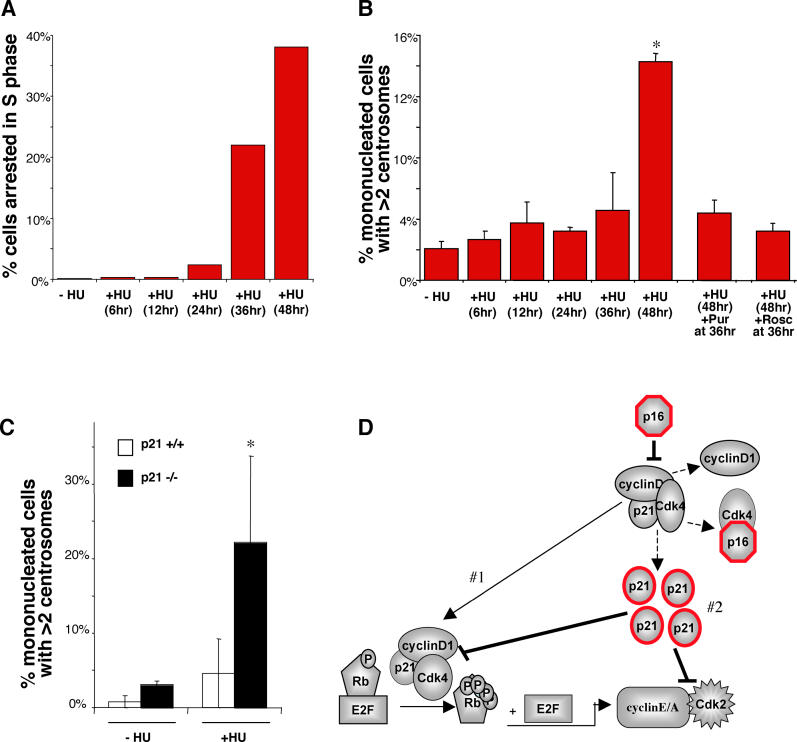
Unregulated Kinase Activity Is Responsible for the Uncoupling of the Centrosome Duplication and DNA Replication Cycles (A and B) vHMECs (RM9 [17 PD], RM16 [20 PD]) that were untreated (−HU) or treated for 6, 12, 24, 36, and 48 h with HU (+HU) were analyzed by (A) flow cytometry of PI and BrdU incorporation (% cells arrested in S phase) and (B) confocal microscopy for mononucleated cells with more than two centrosomes. The kinase inhibitors purvalanol A (Pur) and roscovitine (Rosc) were added following the addition of HU for 36 h and incubated in the presence of HU for 12 h (B). (C) Fibroblasts that express p21 (p21^+/+^) and that have p21 knocked out (p21^−/−^) were analyzed by confocal microscopy for mononucleated cells with more than two centrosomes. Centrosome number was determined by immunocytochemistry with an antibody recognizing the centrosome associated γ-tubulin protein. Centrosome number analysis included at least 200 cells per sample (excluding binucleated cells). *Statistical significance (*p* < 0.005) based on comparison of −HU and +HU experiments. (D) Model of two mechanisms by which p16^INK4a^ can mediate Cdk2 activity. The p16^INK4a^ protein inhibits the G1 kinase activity of the cyclinD1-Cdk4 complex by binding to Cdk4 and preventing its association with cyclinD1, thereby preventing transcriptional activation of the Cdk2 binding partners cyclin E/A (Mechanism 1). The p21 kinase inhibitor is required for assembly of and stabilization of the cyclinD1-Cdk4 complex. In the presence of p16^INK4a^, the cyclinD1-Cdk4 complex is disrupted, causing p21 to be released. When p21 accumulates to a critical threshold, it can bind to and inhibit the late G1 and S phase kinase activity of the cylinE/A-Cdk2 complex (Mechanism 2).

Next, we tested the hypothesis that p16^INK4a^ prevents centriole pair splitting through the regulation of Cdk2 activity. vHMECs were arrested in S phase with HU for various times (0, 6, 12, 24, 36, and 48 h) to determine the time-course of S phase arrest and to determine when cells acquire more than two centrosomes following treatment with HU (Figure 8). Results demonstrate that S phase arrest was achieved at 36 h (Figure 8A). In addition, the results reveal that vHMECs acquire more than two centrosomes at 48 h, 12 h after the cells become arrested in S phase (Figure 8B). To assess if Cdk2 activity is required for acquisition of more than two centrosomes, the Cdk2 inhibitor purvalanol A or roscovitine was added (10 μM) to vHMECs that had been exposed to HU for 36 h, incubated for an additional 12 h, and analyzed for the number of centrosomes per cell (Figure 8B). vHMECs did not acquire more than two centrosomes following HU exposure in the presence of purvalanol A and roscovitine (Figure 8B). Purvalanol A and roscovitine can individually inhibit more than one kinase; however, these two chemical inhibitors have overlapping specificity for Cdk2 inhibition (known to normally be active during S phase) [33]. Therefore, results demonstrating that both inhibitors prevent generation of more than two centrosomes strongly implicate a specific role for regulation of Cdk2 activity in uncoupling of the centrosome duplication and DNA replication cycles. These results suggest that p16^INK4a^ prevents centrosome dysfunction through regulation of Cdk2 activity.

Expression of p16^INK4a^ has been shown to regulate Cdk2 activity and consequently centrosome biology through both pRb (retinoblastoma)-dependent and pRb-independent mechanisms (see model, Figure 8D). First, p16^INK4a^ acts through a pRb-dependent pathway via binding to Cdk4 and inhibition of the cyclin D1/Cdk4 complex. In the absence of active cyclin D1/Cdk4 complex, pRb remains associated with E2F, thereby inhibiting transcriptional activation of the Cdk2 binding partners, cyclin E and A (Figure 8D, #1). Alternatively, acting through a pRb-independent pathway, expression of p16^INK4a^ and its disruption of the association of the cyclin D1/Cdk4 complex have also been shown to directly inhibit Cdk2 activity through a second mechanism. The Cdk inhibitor p21 has been shown to be an assembly factor for the cyclin D1/Cdk4 complex. Therefore, p16^INK4a^-mediated dissociation of the cyclin D1/Cdk4/p21 complex results in release of p21. When unbound p21 accumulates to a certain threshold, it binds to and inactivates the cyclin E/A-Cdk2 complex (Figure 8D, #2) [34,35].

To test the proposed pRB-independent model, we used normal human diploid fibroblasts (HDFs) that have p21 inactivated through targeted homologous recombination (p21^−/−^) [36]. If p16^INK4a^-dependent centrosomal dysfunction is working through this pathway, we anticipate that p21^−/−^ cells would phenocopy loss of p16^INK4a^. Following HU exposure, an average of 22% of the HDFs (p21^−/−^) had more than two centrosomes, similar to the number generated in HDFs lacking functional p16^INK4a^ (NHF p16^INK4a^ –shRNA). In contrast, an average of 3% of the HU-exposed HDFs (p21^+/+^) contained more than two centrosomes (Figure 8C). HDFs (p21^−/−^) had a statistically significant increase in the percentage of cells with more than two centrosomes (*p* < 0.005) as compared to untreated controls.

## Discussion

### Supernumerary Centrosomes Lead to Generation of Aneuploid Daughter Cells

Theodor Boveri hypothesized over 100 years ago that multipolar mitoses were responsible for aneuploidy [6,7]. Previous reports identifying more than two centrosomes in premalignant and malignant cancers and the correlation with aneuploidy provided support for Boveri's hypothesis [8–11]. However, these correlations do not definitively demonstrate that centrosome abnormalities drive genomic instability [12]. In contrast to the majority of studies that use tissue culture cell lines, we looked at genomically intact cells with normal numbers of centrosomes, generated more than two centrosomes in these cells, and subsequently demonstrate that they accumulate multipolar mitotic spindles and an aneuploid karyotype. Cells with supernumerary centrosomes were shown to divide into two nuclei or more than two nuclei. Quantitation revealed that both events resulted in unequal segregation of the DNA. The fold differences observed when cells with supernumerary centrosomes divide into two nuclei likely reflect the unequal segregation of one to two chromosomes (aneuploidy), an interpretation supported by the corresponding karyotypic analysis of these cells. When the cells divide into more than one nucleus, often two or more of the nuclei remain together to form one cell, representing a mechanism for the formation of binucleated and multinucleated cells. These results strongly suggest that supernumerary centrosomes can indeed cause aneuploidy (genomic instability).

### p16^INK4a^ Plays a Critical Role in Centrosome Biology

Our results described a new function of p16^INK4a^ and further demonstrated the importance of p16^INK4a^ in the maintenance of normal cellular properties. In this study, p16^INK4a^ activity was found to be both necessary and sufficient to prevent centriole pair splitting. The p16^INK4a^-dependent centrosome dysfunction was rapidly unmasked under conditions that transiently inhibit DNA synthesis and was gradually unmasked during standard growth in culture. As vHMECs proliferate in culture, they are increasingly exposed to internal signals (e.g., replicative stress, endogenous DNA damage, etc.) and external signals (e.g., oxidative stress, etc.) leading to cell cycle checkpoint responses. We hypothesize that these signals result in transient inhibition of DNA synthesis, thereby providing the opportunity for vHMECs to accumulate greater than two centrosomes as they mature in culture.

We provide evidence that the p16^INK4a^-dependent centrosome dysfunction results from unregulated Cdk2 activity. As described, p16^INK4a^ can regulate Cdk2 activity through both pRb-dependent and pRb-independent mechanisms (see proposed model, Figure 8D) [34,35]. The HeLa cell line contains integrated human papillomavirus (HPV18) and therefore has defective pRb and p53 functions/signaling. Paradoxically, despite loss of these functions, HeLa cells do not have centrosome dysfunction following HU exposure. The shRNA against p16^INK4a^ should have no affect on coupling in HeLa cells if the coupling is a pRb-mediated event. Therefore, our demonstration that HeLa cells become uncoupled when p16^INK4a^ expression is suppressed suggests that p16^INK4a^ is working through a pRb-independent pathway. As described, a pRb-independent pathway by which p16^INK4a^ can regulate Cdk2 activity works through regulation of the bioavailability of the Cdk inhibitor p21 (see model, Figure 8D). Despite HPV-compromised p53 function, HeLa cells are able to induce p21 expression following a stress response [37], and this increased bioavailability of p21 in HeLa cells may explain their coupled centrosome duplication and DNA replication cycles. An important caveat in studying signaling pathways in established tumor cell lines is the reality that they contain a plethora of mutations with unknown consequences on the pathways studied. Therefore, we assessed the pRb-independent model in normal human diploid cells with intact signaling pathways. Our data provide evidence that p16^INK4a^ can act independent of pRb to regulate Cdk2 activity and to couple the DNA replication and centrosome duplication cycles. These experiments also provide evidence that the inactivation of p16^INK4a^ will engender phenotypes beyond those that are induced simply by inactivation of pRb.

### Transient Inhibition of DNA Replication, Often Observed with Chemotherapeutic Agents, Is Needed to Unmask Centrosomal Dysfunction

A previous report examined normal human oral keratinocytes engineered to inhibit p16^INK4a^ function through overexpression of Cdk4 [38]. When examined for centrosomal abnormalities, mononucleated cells with more than two centrosomes are seen in 1% of the parent population and 3% of the Cdk4-overexpressing population. The authors conclude that disruption of the pRb checkpoint through inactivation of p16^INK4a^ does not necessarily lead to centrosomal-associated abnormalities or genomic instability. Our results in the HMEC system are consistent with this report. In data presented here, the removal of p16^INK4a^ activity (by expression of shRNA) did not, in and of itself, generate more than two centrosomes. The p16^INK4a^-suppressed cells required the transient inhibition of DNA synthesis to unmask the uncoupled cycles and generate more than two centrosomes. Conditional phenotypes such as this are a hallmark of checkpoint controls, which are, as originally defined, not essential for viability but are only unmasked under specific conditions [39]. These data suggest that under physiological conditions in which proliferation is not occurring (G0) or conditions where it proceeds in an uninterrupted fashion, p16^INK4a^-suppressed cells would retain normal centrosome numbers. However, under conditions that trigger inhibition of the DNA replication cycle, the absence of p16^INK4a^ allows the centrosome duplication cycle to proceed, resulting in an abnormal numbers of centrosomes. Further insight into the mechanism by which p16^INK4a^ prevents generation of more than two centrosomes during cell cycle arrest was gained from the observation that, in the absence of p16^INK4a^, the duplicated centrosomes progress into a second round of centrosome duplication that ceases at the stage of centriole pair splitting. These results suggest that p16^INK4a^ functions to prevent centriole splitting during prolonged S phase arrest.

In addition, our observations have important clinical ramifications. Previously, we demonstrated that human mammary epithelial cells lacking p16^INK4a^ function exist in vivo in disease-free women [40]. Since HU is currently used as a chemotherapeutic agent, treatment of patients with an HU or other chemotherapeutic chemicals such as actinomycin or 5-fluorouracil that arrest p16^INK4a^-deficient cells (uncoupled) in S phase may perpetuate the disease by creating cells with more than two centrosomes and subsequent aneuploidy [41].

### Contribution of Centrosomal Dysfunction to Early Events in Cancer

The importance of p16^INK4a^ in tumorigenesis is highlighted by findings that p16^INK4a^ point mutations cosegregate with tumor susceptibility in familial melanoma and pancreatic cancer. Aneuploidy is an early and frequent characteristic of these cancers [42]. In addition, genetic and epigenetic alterations of p16^INK4a^ are frequently seen in many types of cancer [43]. For example, studies on the earliest stages of multiple myeloma show that the majority of cells within the early lesions contain hypermethylated (silenced) p16^INK4a^ and that the first genomic abnormality detected is aneuploidy [44].

These studies document a subpopulation of HMECs, vHMECs, that have uncoupled centrosome duplication and DNA replication cycles and accumulated centrosomal abnormalities. vHMECs acquire many phenotypes that are seen in premalignant lesions, including expression changes (cyclooxygenase-2), genomic instability, telomeric dysfunction, and, as described here, centrosome abnormalities [22,45]. A distinguishing characteristic of vHMECs, p16^INK4a^ promoter hypermethylation and subsequent silencing of protein expression, has been demonstrated in histologically normal mammary tissue from a substantial fraction of healthy, disease-free women with no predisposition to breast cancer [40]. p16^INK4a^ silencing is also a distinguishing characteristic of several types of stem cells. Under stress conditions [46,47], p16^INK4a^-silenced cells (vHMECs or stem cells) have a proliferative advantage and would be expected to expand in number at the expense of the p16^INK4a^-positive cells (HMECs). Internal and external insults would result in DNA damage and act to signal inhibition of the DNA replication cycle. As described here, if p16^INK4a^-silenced vHMECs are exposed to conditions that result in the transient inhibition of the DNA replication cycle, these cells would acquire more than two centrosomes, which would then result in abnormal polarity and multipolar mitoses and subsequently result in the generation of aneuploid daughter cells (characteristics of premalignant lesions). Hence, the loss of p16^INK4a^ function would allow the cells to grow under conditions where they would ordinarily be arrested and result in accumulation of genomic abnormalities during expansion. Gain or loss of appropriate genomic material (aneuploidy) would provide vHMECs or stem cells with the necessary proproliferation and antiapoptotic mechanisms needed for tumorigenesis.

## Materials and Methods

### Cells and cell culture

Isolation of HMECs and vHMECs and routine cell culture in modified MCDB 170 (MEGM; BioWhittaker, Walkersville, Maryland, United States) media was performed as previously described [22]. HMF were isolated and cultured in DME H-21 with 10% fetal calf serum. We used HMECs, vHMECs, and HMFs from RM specimens from five different individuals: RM9, RM15, RM16, RM21, and RM26. NHFs were isolated from human newborn foreskin. PDs were calculated using the equation, PD = log *(A/B)*/log2, where *A* is the number of cells collected and *B* is the number of cells plated. HeLa and Chinese hamster ovary (CHO) cells were obtained from the American Type Culture Collection (ATCC, Manassas, Virginia, United States) and cultured in DME H-21 with 10% fetal calf serum.

### Immunocytochemistry and confocal microscopy

For immunofluorescence staining, HMECs were grown on glass coverslips and fixed for 10 min with −20 °C methanol. Cells were permeabilized with 0.01% Triton X-100 prior to incubation with the primary antibody. Blocking and antibody dilution buffer was PBS containing 5% goat serum (GIBCO-BRL, San Diego, California, United States), 0.1% bovine serum albumin (Sigma, St. Louis, Missouri, United States), 0.1% fish skin gelatin (Sigma), and 1% glycerol (Fisher Scientific, Pittsburgh, Pennsylvania, United States). A monoclonal antibody recognizing γ-tubulin (clone GTU-88; Sigma) was used at 1 μg/ml to immunostain the pericentriolar material of the centrosome. An antibody recognizing centrin (clone 20H5) was used at 1:1,000 dilution to immunostain the centrioles of the centrosome. The centrin antibody was kindly provided by Dr. J. Salisbury. A monoclonal antibody recognizing γ-tubulin (clone DM1A; Sigma) was used at 1:1,000 dilution to immunostain microtubules. A monoclonal antibody recognizing p16^INK4a^ (clone Ab-7; NeoMarkers, Lab Vision Corp., Fremont, California, United States) was used at 1:100 dilution. Secondary antibody used for detection of the anti–γ-tubulin, the anti-centrin, the anti–γ-tubulin, and the anti-p16INK4a antibodies was a fluorescein isothiocyanate (FITC)-conjugated sheep F(ab′)2 fragment to mouse IgG (whole molecule) (ICN/Cappel Biomedicals, Costa Mesa, California, United States) or a tetramethylrodamine (TRITC)-conjugated anti-mouse IgG (Southern Biotechnologies, Birmingham, Alabama, United States). DNA was stained using DAPI (Vector Technologies, Milwaukee, Wisconsin, United States) or ToPro3 (Molecular Probes, Eugene, Oregon, United States). Glass coverslips were mounted to slides with anti-fade mounting media (Vectashield; Vector Technologies). Samples were analyzed on a Zeiss 510 LSM confocal microscope using an Argon/2 laser (excitation 488 nm), a HeNe2 laser (excitation 633 nm), and a titanium:sapphire dual-photon laser (tuned to 790 nm). Standard deviation between separate experimental trials performed under identical conditions was determined and expressed as error bars when appropriate. Statistical significance was determined by the two-sided Fischer exact test (95% confidence interval).

### Cell cycle analysis

Cells were plated at a density of 2 × 10^3^ cells/cm^2^. Cells were metabolically labeled with bromodeoxyuridine (BrdU) (10 μM, 4 h), trypsinized, and fixed with 70% ethanol. Nuclei were isolated and stained with PI and FITC-conjugated anti-BrdU antibodies (Becton-Dickinson, Palo Alto, California, United States), as previously described [22]. Flow cytometry was performed on an FACSort (Becton-Dickinson). All analyzed events were gated to remove debris and aggregates.

### DNA quantification

For quantitative analysis of DNA content, HMECs and vHMECs were grown on glass coverslips and fixed for 10 min with −20 °C methanol. Immunocytochemistry for γ-tubulin was performed as described above. DNA was stained using 3 μg/ml PI and 0.2 mg/ml RNase in PBS for 1 h at room temperature, as previously described. The coverslips were mounted as described and the samples were analyzed on a Zeiss 510 LSM confocal microscope using an Argon/2 laser (excitation 488 nm) and a HeNe1 laser (excitation 543 nm). Quantification of DNA content for each cell was determined using Velocity software (Improvision, Lexington, Massachusetts, United States) to determination the total sum of PI signal (intensity) for each nucleus. Dot-blot analysis (centrosome number versus PI intensity) allowed us to determine whether cells had a 2N-4N DNA content (diploid) or a more than 4N DNA content (polyploidy). Note that cells of 2N DNA content were determined empirically for each sample set by assigning cells with one centrosome as 2N. The cells of 4N and more than 4N DNA content were then extrapolated from this value. Background subtraction of fluorescent signal was performed in areas not containing nuclei.

### Reduction of polyploid population in vHMECs

vHMECs were grown on plastic tissue culture plates, trypsinized, washed, and sorted by FSC versus SSC on an FACSVantage SE (Becton-Dickinson). Cells in the lower FSC and SSC quadrants were gated and sorted for recovery. Low FSC/SSC cells were plated and grown in tissue culture media containing penicillin, streptomycin, and fungicide. Cells were subsequently monitored for DNA content by staining for PI and analyzed by flow cytometry.

### Inhibition of DNA synthesis

Cells were plated onto glass coverslips at a density of 1 × 10^4^ to 2 × 10^4^ cells per well in a 24-well plate or on plastic tissue culture dishes at a density of 2 × 10^3^ cells/cm^2^. HU (Sigma) was dissolved in water and further diluted in cell culture media to generate a final concentration of 4 mM. Cells were exposed to 4 mM HU for 48 h (unless otherwise stated) prior to analysis by immunocytochemistry. HU was applied to cells 1–2 d after initial seeding. Analysis was restricted to mononucleated cells. To minimize variability in our experimental conditions, the untreated controls (−HU) and HU-exposed (+HU) cells for each experiment were always from the same individual of matched passage number. The Cdk2 inhibitors purvalanol A and roscovitine (Sigma) were diluted in cell culture media at a concentration of 10 μM.

### Transient transfection and viral infection of cultured cells

vHMECs were transiently transfected using TransIT-LT1 transfection reagent (Mirus, Madison, Wisconsin, United States) with wild-type p16^INK4a^ in pBabe-puro or the pBabe-puro plasmid alone provided by F. McCormick [48]. We used retrovirus-mediated delivery of constructs encoding EGFP-CETN2 [49], EGFP–γ-tubulin [50], EGFP-H2B (PharMingen, San Diego, California, United States), and an shRNA specific for p16^INK4a^. EGFP-CETN2 and EGFP–γ-tubulin, EGFP alone, and the p16^INK4a^ cDNAs were subcloned into pBabe-puro and the EGFP-H2B was subcloned into pBabe-hygro. The shRNA specific for p16^INK4a^ was expressed from the RNA PolIII–specific U6 promoter similar to the approach previously described [51]. The shRNA encoded inverted repeats of 27 base pairs corresponding to nucleotides 381 to 407 of the human CDKN2A p16^INK4a^ cDNA, separated by an eight-nucleotide spacer [30]. We produced amphotropic retrovirus by transfecting Phoenix-A packaging cells with empty vector, GFP shRNA, and p16^INK4a^ shRNA using LipofectAMINE PLUS reagent (Qiagen, Valencia, California, United States) and harvesting and filtering the virus-containing culture medium 48 and 72 h posttransfection. Transfection frequency was routinely greater than 60%, as determined by parallel transfection of Phoenix-A cells with a plasmid containing a GFP expression cassette (data not shown). Cells were infected by exposing them to virus-containing medium for 4–6 h, each with an intervening 20-h recovery period. At 72–96 h after the first infection, cells were trypsinized and plated in the presence of 4 μg/ml Puromycin (Sigma) or 20 μg/ml Hygromycin (Sigma). Following infection, cells were maintained in medium containing 4 μg/ml Puromycin and/or 20 μg/ml Hygromycin as appropriate. Infection frequencies were routinely in the range of 3% to 10%, as determined by colony formation assays.

### Chromosomal analysis

Metaphase spreads were prepared from cells treated with colcemid (KaryoMAX; GIBCO-BRL, 100 ng/ml for 2 h). We performed standard G-banding karyotypic analysis on at least 50 metaphase spreads for each population described. Metaphase spreads were classified as abnormal if they contained any complement of chromosomes besides 46XX with normal banding patterns.

### Time-lapse image analysis

Time-lapse microscopy was performed using a Zeiss LSM 510 confocal microscope with a plan-apochromat ×63 oil objective (1.4 n.a.). The EGFP–γ-tubulin and EGFP-H2B fusion proteins were excited using a two-photon, titanium:sapphire laser (tuned to 880 nm). During image acquisition, the cells are maintained at a constant temperature of 37 °C with 5% CO_2_ using a heated stage, a stage-mounted incubator, and a CO_2_ regulator (Zeiss). A *z*-stack was acquired every 3–4 min. During image acquisition, the microscope was enclosed with a Plexiglas box custom made with a heater and fan (TX7 Wired Dome assembly; Lyon Electric Company, Chula Vista, California, United States) to maintain a temperature of 32–37 °C to minimize the thermal drift of the microsope. An objective heater (Zeiss) was also used at 37 °C to minimize thermal drift of the objective. Quantification of DNA content for each cell was determined using Velocity software (Improvision) to determination the total sum of EGFP-H2B signal (intensity) for each nucleus. The EGFP-H2B signal of daughter cells was quantified from up to ten time frames per mitotic division of those that resulted from mitotic division with two or more than two centrosomes. The fold difference between the daughter cells (signal intensity of daughter cell 1/signal intensity of daughter cell 2) was determined for each time frame. The fold differences of each time frame were than averaged to determine the average fold difference. Note that EGFP–γ-tubulin signal was not always able to be separated from the EGFP-H2B signal when selecting and quantifying. To ensure that the selection of EGFP–γ-tubulin signal is not contributing significantly to the determined fold differences, we determined the signal intensity of single centrosomes (EGFP–γ-tubulin signal) as compared to a clearly defined nuclei (EGFP-H2B signal). The average total signal intensity of a single centrosome is less than 0.3% of the total signal intensity of a nucleus. Thus, the signal intensity from the EGFP–γ-tubulin would not contribute to give a significant fold difference. Background subtraction of EGFP-H2B signal was performed in areas not containing nuclei. Only cells that were imaged completely within the *x,y,z* plane of the acquisition were used for quantitation. Statistical significance was determined by the two-sided *t*-test (95% confidence interval).

## Supporting Information

Video S1 Time-Lapse Microscopy of vHMECs during Mitosis with a Bipolar Spindle Organized by Two Centrosomes Time-lapse microscopy of early-passage vHMECs (RM18 [13 to 33 PD]) that express EGFP–γ-tubulin (green) and EGFP-H2B (green) during mitotic progression of cells that divide into two nuclei with two centrosomes (+HU → release; bipolar, two centrosomes). The *x* (green arrow), *y* (red arrow), and *z* (blue arrow) position designates the orientation of the cells (lower left corner). The red grid (with unit value in μm placed in the lower right corner) designates the proportional scale. The relative time of each frame is placed in the upper right corner.
(219 KB MOV).

Video S2 Time-Lapse Microscopy of vHMECs during Mitosis with a Pseudo-Bipolar Spindle Organized by More Than Two Centrosomes Time-lapse microscopy of early-passage vHMECs (RM18 [13 to 33 PD]) that express EGFP–γ-tubulin (green) and EGFP-H2B (green) during mitotic progression of cells that divide into two nuclei with more than two centrosomes (+HU → release; pseudo-bipolar, more than two centrosomes). The *x* (green arrow), *y* (red arrow), and *z* (blue arrow) position designates the orientation of the cells (lower left corner). The red grid (with unit value in μm placed in the lower right corner) designates the proportional scale. The relative time of each frame is placed in the upper right corner.
(244 KB MOV).

Video S3 Time-Lapse Microscopy of vHMECs during Mitosis with a Multibipolar Spindle Organized by More Than Two Centrosomes Time-lapse microscopy of early-passage vHMECs (RM18 [13 to 33 PD]) that express EGFP–γ-tubulin (green) and EGFP-H2B (green) during mitotic progression of cells that divide into greater than two nuclei with more than two centrosomes (+HU → release; multipolar, with more than two centrosomes). The *x* (green arrow), *y* (red arrow), and *z* (blue arrow) position designates the orientation of the cells (lower left corner). The red grid (with unit value in μm placed in the lower right corner) designates the proportional scale. The relative time of each frame is placed in the upper right corner.
(97 KB MOV).

### Accession Numbers

The GenBank (http://www.ncbi.nlm.nih.gov/Genbank) accession numbers for proteins discussed in this paper are p16^INK4a^ (NM_000077), human centrin 2 (NM_004344), H2B (X00088), γ-tubulin (NM_001070), and p21 (NM_078467).

## Abbreviations

BrdU: bromodeoxyuridine
Cdk: cyclin-dependent kinase
EGFP-CETN2: enhanced green fluorescent protein–human centrin 2
FSC: forward scatter
GFP: green fluorescent protein
HDF: human diploid fibroblast
HMEC: human mammary epithelial cell
HMF: human mammary fibroblast
HU: hydroxyurea
NHF: newborn-derived foreskin fibroblast
PD: population doubling
PI: propidium iodide
RM: reduction mammoplasty
shRNA: short hairpin RNA
SSC: side scatter
vHMEC: variant human mammary epithelial cell
